# PG2 for patients with acute spontaneous intracerebral hemorrhage: a double-blind, randomized, placebo-controlled study

**DOI:** 10.1038/srep45628

**Published:** 2017-03-31

**Authors:** Chun-Chung Chen, XianXiu Chen, Tsai-Chung Li, Hung-Lin Lin, Yen-Tze Chu, Han-Chung Lee, Yu-Kai Cheng, Der-Cherng Chen, Shiu-Chiu Tsai, Der-Yang Cho, Ching-Liang Hsieh

**Affiliations:** 1 School of Medicine, College of Medicine, China Medical University, Taichung 40402, Taiwan; 2 Department of Neurosurgery, China Medical University Hospital, Taichung 40447, Taiwan; 3 Stroke Center, China Medical University Hospital, Taichung 40447, Taiwan; 4 Department of Public Health, China Medical University, Taichung 40402, Taiwan; 5 Graduate Institute of Biostatistics, China Medical University, Taichung 40402, Taiwan; 6 Department of Neurosurgery, Tainan Municipal An-Nan Hospital, Tainan 70965, Taiwan; 7 Graduate Institute of Immunology, China Medical University, Taichung 40402, Taiwan; 8 Department of Chinese Medicine, China Medical University Hospital, Taichung 40447, Taiwan; 9 Graduate Institute of Integrated Medicine, College of Chinese Medicine, China Medical University, Taichung 40402, Taiwan; 10 Graduate Institute of Acupuncture Science, College of Chinese Medicine, China Medical University, Taichung 40402, Taiwan; 11 Research Center for Chinese Medicine and Acupuncture, China Medical University, Taichung 40402, Taiwan

## Abstract

PG2 is an infusible polysaccharide extracted from *Astragalus membranaceus*, which is a Chinese herb traditionally used for stroke treatment. We investigated the effect of PG2 on patients with spontaneous acute intracerebral hemorrhage (ICH). A total of 61 patients with acute spontaneous ICH were randomized to either the treatment group (TG, 30 patients), which received 3 doses of PG2 (500 mg, IV) per week for 2 weeks, or the control group (CG, 31 patients), which received PG2 placebo. At 84 days after PG2 administration, the percentage of patients with a good Glasgow outcome scale (GOS 4–5) score in the TG was similar to that in the CG (69.0% vs. 48.4%; p = 0.2). The percentage of good mRS scores (0–2) in the TG was similar to that in the CG (62.1% vs. 45.2%; p = 0.3). In addition, no significant differences were seen when comparing differences in the C-reactive protein, erythrocyte sedimentation rate, interleukin-6 (IL-6), IL-1β, tumor necrosis factor-α, and S100B levels between baseline and days 4, 7, and 14 after PG2 administration (all p > 0.05). The results are preliminary, necessitating a more thorough assessment.

Hypertensive spontaneous intracerebral hemorrhage (ICH) is responsible for 10–15% of strokes, and is their most lethal cause, with a one-month mortality rate of 30–50%^1^. ICH is also associated with high levels of disability, with only 20% of survivors being functionally independent at 6 months and 36% of survivors remaining moderately to severely disabled at discharge^2^. Therefore, treatments that enhance the recovery of neurologic function are necessary. *Astragalus membranaceus* (AM) is a Chinese herb used extensively in China as a traditional treatment to facilitate recovery after a stroke. Our previous studies have demonstrated enhanced recovery of neurologic function in patients with acute hemorrhagic stroke who received AM. It is hypothesized that AM either reduces inflammatory response or reduces perihematomal edema^3^,^4^. AM can be administered only orally; by contrast, PG2, a polysaccharide extract from AM, has been approved for intravenous (IV) use in treating cancer-related fatigue in Taiwan^5^. Several preclinical studies have shown that PG2 stimulates the secretion of hematopoietic growth factors in activated human peripheral blood mononuclear cells^6^, enhances proliferation and maturation of peripheral blood cell progenitors in mitomycin C-treated mice^7^, and supports hematopoiesis in long-term bone marrow cultures^8^. These results indicate that PG2 can stimulate immunity and decrease inflammation.

The basal ganglion is the most common location of hypertensive spontaneous ICH, followed by the subcortical white matter, and then the cerebellum or pons^9^. Hematomas that extend to the ventricles, that are in a deep location, and that have a large mass and volume are associated with poor outcomes in patients with medium and large supratentorial ICH^10^. Hematoma size can affect the six-month survival rate after stroke. For instance, patients with hematomas with a volume of <29 mL have a survival rate of 64%, whereas patients with hematomas with a volume of >60 mL have a survival rate of 15%^11^. Both hematoma size and brain edema, which are caused by the breakdown of the brain–blood barrier (BBB), can cause cerebral ischemia, resulting in primary brain damage. In addition, several factors may induce secondary brain damage: (1) the presence of heme and iron, from erythrocyte lysis and free hemoglobin, which can incite reactive oxygen species production; (2) the disruption of the BBB, which can cause leucocyte infiltration; and (3) the presence of intracerebral blood, which can induce microglial activation, systemic immune cell infiltration, and the generation of proinflammatory cytokines such as tumor necrosis-*α* (TNF-α), interleukin-1beta (IL-1β), interleukin-6 (IL-6), and other chemokines^12^,^13^,^14^. Inflammatory response thus plays a critical role in the pathophysiology of ICH.

We hypothesize that the anti-inflammatory response induced by PG2 can counter the inflammatory response induced by ICH, which in turn enhances the recovery of neurologic function. Therefore, in the present preliminary double-blind, randomized, placebo-controlled study, we evaluated the effect of PG2 on patients with acute spontaneous ICH.

## Materials and Methods

### Subjects

Patients were recruited from the neurosurgery and emergency departments of China Medical University Hospital, Taichung City, Taiwan, from March 30, 2011, to June 14, 2013. All patients were recruited within 24 h of ICH onset. The study complied with the ethical principles of clinical trials as dictated in the Declaration of Helsinki. The protocol was approved by the Institutional Review Board of China Medical University Hospital (IRB: DMR100-IRB-005; NIH registration NCT 01325233, March 28, 2011), and informed consent was provided by patients prior to inclusion in the study. The study also abided by the International Conference on Harmonization and Good Clinical Practice guidelines. The inclusion criteria were as follows: (1) both female and male patients, (2) between the ages of 30 and 80 years, (3) admission within 24 h of ICH onset, (4) first incidence of hemorrhagic stroke with the hematoma located in the putamen, and (5) a signed informed consent by the patient or their legal representative. The exclusion criteria were as follows: (1) recent thrombolysis treatment, (2) history of previous stroke, (3) full-dose or long-term anticoagulation therapy, (4) hemorrhagic stroke but the location was not the putamen, (5) coexisting systemic diseases such as terminal cancer, renal failure, liver cirrhosis, severe dementia, or psychosis, (6) participation in another clinical trial within the last three months, (7) pregnancy or lactation, and (8) planned surgical evacuation of hematoma.

### Preparation of PG2

The investigational drug PG2 (PhytoHealth Corp., Taiwan) was extracted from AM as previously described^6^,^7^,^8^. PG2 is an IV injectable extract of AM polysaccharide approved by the Taiwan Food and Drug Administration for cancer-related fatigue (Drug No. 054853). Prior to administration, this polysaccharide mixture was formulated as a sterile powder. The PG2 sterile powder was stored in the clinical trial pharmacy department of China Medical University Hospital. The pharmacist used a vial of PG2 (500 mg) reconstituted with 10 mL normal saline and shaken thoroughly until completely dissolved. It was then injected into a bag of normal saline (490 mL) and mixed well prior to intra-venous (IV) infusion at 150–200 mL/h. The appearance and odor of PG2 injection was not different from that of a normal saline injection based on naked sensory assessment.

### Design and Sample Size

The present study was a preliminary, single-center, double-blind, placebo-controlled, randomized phase II/III study. Sample size was calculated according to the hypothesis that PG2 can increase a patient’s good outcome score (mRS score ≤2) percentage in ten dimensions. We further hypothesized that the increase in mRS scores would be evident when comparing mRS scores at the baseline (prior to PG2 administration) to those at one week (W1), four weeks (W4) and twelve weeks (W12) after PG2 administration. According to our previous results^3^, a sample size of 46 patients (23 per group) was necessary to achieve a statistical power of 90%. Assuming a patient follow-up rate of 0.8, at least 58 patients were required for this study.

### Randomization and Grouping

Subjects were randomly assigned to either the treatment group (TG) or the control group (CG). The statistical center of China Medical University applied a block randomization with a block size of two or four by using computer generated random numbers. The random numbers were placed in a sealed envelope that was then sent to the manufacturer of PG2. The manufacturer packed and labelled the study drug package with a random number, and then forwarded it to the clinical trial pharmacy. The labelled package was managed by a specific pharmacist. After screening and meeting the inclusion and exclusion criteria, the subjects signed the informed consent form. The coordinator then called the pharmacist to enroll a new subject. The pharmacist then dispensed the drug according to the sequence number. In the TG, the subjects received an IV injection of PG2 (500 mg in 500 mL saline) once daily 3 days per week for 2 weeks after starting treatment within 24 hours of stroke onset. All patients received the standard treatment according to the Guidelines for the Management for Spontaneous Intracerebral Hemorrhage (American Stroke Association, 2010)^15^. The CG patients received an IV injection of PG2 placebo (500 mL, normal saline). Thus, the study team could not distinguish the placebo from true PG2. The patients, investigators, coordinator, and study nurse were blinded.

### Outcome measures

The primary outcome measures were the change in the percentage of good Glasgow outcome scale (GOS) and/or Modified Rankin scale (mRS) scores after PG2 treatment. Good outcome scores were defined as GOS scores of 4–5 and mRS scores of 0–2. Bad outcome scores were defined as GOS scores of 1–3 and mRS scores of 3–5. The secondary outcome measures included changes in C-reactive protein (CRP), erythrocyte sedimentation rate (ESR), interleukin-6 (IL-6), interleukin-1β (IL-1β), tumor necrosis factor-α (TNF-α), and S100B calcium-binding protein B (S100B) after PG2 treatment.

A certified clinical trial coordinator blinded to the groups assessed the GOS, mRS scores at baseline (B), one week (7 ± 1 days; W1), four weeks (28 ± 4 days; W4) and 12 weeks (84 ± 10 days; W12) after PG2 administration. CRP, ESR, IL-6, IL-1β, TNF-α, and S100B were assessed at B and at Days 4 (D4), 7 (D7), and 14 (D14) after PG2 administration.

### CRP, ESR, IL-6, IL- IL-1β, TNF-α, and S100B measurement

Four mL of blood was drawn from the cubital vein at the baseline and at days 4, 7, and 14 after PG2 administration. The blood was divided into two tubes containing 2.0 mL of blood each. One tube was sent to the laboratory department of China Medical University Hospital for CRP and ESR measurements. The other tube was sent for IL-6, IL-1β, TNF-α, and S100B measurements to the neuroscience laboratory of China Medical University. The samples were centrifuged for 10 min at 3600 rpm, and the supernatant was immediately stored at −20 °C until analysis. The IL-1β, IL-6, TNF-α, and S100B levels were measured using commercial enzyme-linked immunosorbent assay (ELISA) kits (IL-1β, IL-6 and TNF-α; eBioscience, USA; S100B, Millipore, USA) and an ELISA reader (BioTek Instruments, USA). No cross-reactivity or interference with other related interleukins was observed. The data were represented in picograms per milliliter, and all assays were performedduplicate. The ELISA kit sensitivities for IL-1B, IL-6, TNF-a, and S100B are 0.3 pg/mL, 0.92 pg/mL, 2.3 pg/mL, and 2.7 pg/mL.

### Statistical Analysis

Baseline variables were compared using a two-group t-test for continuous variables (e.g., age) and a chi-squared (χ^2^) test for categorical variables (e.g., gender). Intention-to-treat analysis was used. The efficacy variables of the two groups were compared at B, W1, W4, and W12, respectively. The two-sample t-test was used separately for each comparison. To consider multiple testing, p-values were reported using false discovery rate method, a linear step up adjustment for false discovery rate. To allow for the possibility of nonnormal distribution, the nonparametric Mann–Whitney test was performed. All analyses were performed using SAS version 9.2 (SAS Institute Inc., Cary, NC); *p* ≤ 0.05 was considered statistically significant.

## Results

### Baseline demographic data characteristics

**Figure 1:**
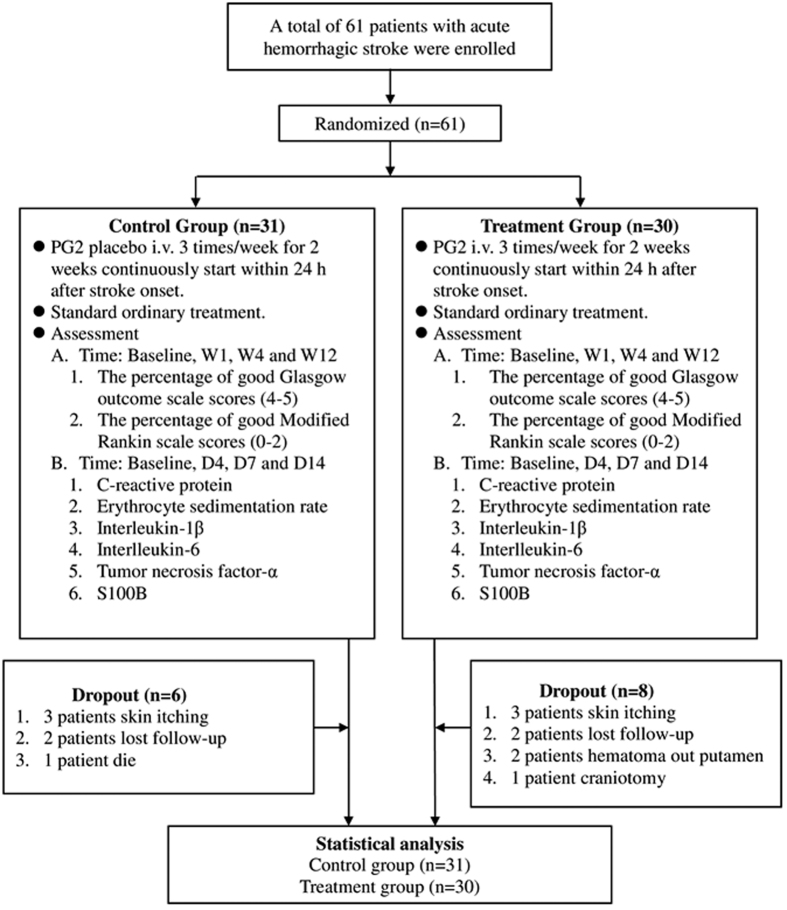
Flowchart. Baseline: prior to PG2 treatment; W1: 7 days after PG2 treatment; W4: 28 days after PG2 treatment; W12: 84 days after PG2 treatment; D4: Day 4 after IV PG2 injection; D7: Day 7 after IV PG2 injection; D14: Day 14 after IV PG2 injection.

Sixty-one patients with acute spontaneous ICH were recruited into this study and were randomized to either the CG (31 patients) or the TG (30 patients). Six patients dropped out in the CG: three dropped out due to pruritus, two were lost to follow-up, and one died of congestive heart failure. Eight patients dropped out in the TG: three dropped out due to pruritus, two because of the presence of hematomas not located in the putamen, one owing to having a craniotomy performed, and two were lost to follow-up. The remaining 47 patients (25 in the CG and 22 in the TG) completed the trial (Fig. 1). Baseline characteristics—namely gender, age, body temperature (BT), blood pressure (including systolic blood pressure and diastolic blood pressure), atrial fibrillation, ischemic heart disease, diabetic mellitus, use of statins and antiplatelet medications, Glasgow coma scale score, intraventricular hemorrhage (IVH), hematoma size and location (i.e., right or left hemisphere)—in the CG and TG did not differ significantly (Table 1).

### Effect of PG2 on the percentage of good and bad GOS and mRS scores in patients with acute spontaneous ICH

The percentage of good and bad GOS scores at B, W1, W4 and W12 did not differ significantly between CG and TG (all *p* > 0.05; Table 2).

The percentage of good and bad mRS scores at B, W1, W4, and W12 did not differ significantly between the CG and the TG (all *p* > 0.05; Table 2).

### Effect of PG2 on CRP, ESR, IL-6, IL-1β, TNF-α, and S100B in patients with acute spontaneous ICH

The CRP, ESR, IL-6, IL-1β, TNF-α, and S100B levels at B, D4, D7, and D14 did not differ significantly between the CG and TG (all *p* > 0.05; Table 3). No significant differences were seen when comparing differences in the CRP, ESR, IL-6, IL-1β, TNF-α, and S100B levels between B and D4, D7, and D14 (all *p* > 0.05; Table 3).

Regarding the area under the curve measures, the percentage of log CRP, ESR, IL6, IL-1β, TNF-α, and S100B from baseline to D14 after PG2 treatment were not significantly different between the CG and the TG (all *p* > 0.05; Table 4).

### Adverse events

The adverse events encountered in the present study included headache, cough, fever, constipation, skin puritis, and urinary problems. These adverse events were not significantly different for CG and TG (all *p* > 0.05; Table 5). One patient died of congenital heart failure because of myocardial infarction induced by factors unrelated to the study medications.

## Discussion

The present study demonstrated that there was no significant difference between the CG and the TG regarding the percentages of good GOS and mRS scores at B, W1, W4, and W12. The CRP, ESR, IL-6, IL-1β, TNF-α, and S100B levels at B, D4, D7, and D14 did not differ significantly between the CG and TG. No significant differences were seen when comparing differences in the CRP, ESR, IL-6, IL-1β, TNF-α, and S100B levels between B and D4, D7, and D14. Taken together, these results suggest administration of PG2 for 2 weeks would not increase the percentage of good GOS and mRS scores, and that also not produced any anti-inflammatory properties. The Surgical Trial in Intracerebral Hemorrhage group found no significant difference in neurological function and mortality rates between patients treated with early surgery and those who received conservative treatment^16^,^17^. Physiological responses to hematomas and hematoma degradation products may contribute to the development of inflammatory reactions^18^,^19^,^20^. Inflammation begins immediately after hematoma formation, and increasing evidence suggests that inflammation is a crucial contributor to ICH-induced secondary brain injury^19^. The mechanisms of ICH-induced brain damage mediated by inflammation are complex and involve multiple signaling pathways^19^. Anti-inflammatory medications provide potential strategies for treating ICH. Preclinical experiments have reported that inhibition of the inflammatory response is an effective approach in the treatment of ICH^21^. ICH causes perihematomal edema, which increases the mass effect and intracranial pressure (ICP) and may exacerbate brain damage or even lead to cerebral herniation^22^,^23^. Increased ICP may reduce microcirculation through mechanical compression^24^,^25^, and edema may change osmotic gradients and disrupt the BBB resulting in direct toxicity to the neurons and glia^26^,^27^. Many studies have reported that increased water content, also called perihematomal edema, is involved in apoptosis and necrosis after ICH^28^,^29^,^30^. Studies have also shown that antioxidant therapy can decrease brain edema^31^. AM polysaccharides increased splenic lymphocyte proliferation and IL-2 levels in rats with gastric neoplasia, indicating that they play an anti-inflammatory and immune modulating role^32^. They also decrease CD40 expression^32^,^33^, which has both inflammatory and anti-inflammatory actions in renal proximal tubular epithelial cells, as well as regulating these two processes^34^. In addition, AM polysaccharides have antioxidant properties, scavenging superoxide anions and hydroxyl radicals^33^.

Serum CRP levels increase in response to inflammation and following IL-6 secretion^35^. CRP levels >10 mg/L are predictive factors for early hematoma growth and worsening neurologic function in patients with acute spontaneous ICH^36^. ESR is a nonspecific test in which an increase in ESR suggests the presence of inflammation. ESR is often used as a laboratory indicator to assess clinical disease in patients with polymyalgia rheumatic and giant cell arteritis^37^. Both TNF-α and IL-1β are proinflammatory cytokines that play an important role in inflammation and BBB disruption after ICH. TNF-α is secreted from neurons and activated microglia and astrocytes. IL-β is produced and secreted by activated microglia and astrocytes. In a porcine model of ICH, TNF-α and IL-1β expression measurements at 4 h after ICH revealed decreased TNF-α levels. However, the IL-1β levels did not decrease even 24 h after ICH^38^. TNF-α antibodies, such as CNTO 5048, reduced inflammation and improved functional outcome in a murine ICH model^39^. By contrast, TNF-α was found to have anti-inflammatory effects through negatively regulating inflammation^40^. Thus, TNF-α plays a dual role in the regulation of inflammation. In a murine model of hypertensive ICH, IL-1β levels correlated positively with brain edema^41^. IL-6 levels were elevated after ICH and correlated with blood volume and the mass effect^42^. IL-6 has both a proinflammatory and an immunomodulatory role, and may be proinflammatory after acute stroke; however, it is not involved in brain damage^43^. In addition, IL-6 has both anti-inflammatory and immunosuppressive properties^44^. S100B is a Ca^2+^ binding protein present in the central nervous system, mainly in glial cells. Serum levels rise after ICH, and S100B levels have a positive correlation with hematoma volume and can predict early neurologic deterioration and unfavorable outcomes 3 months after acute spontaneous ICH^45^. Serum S100B levels increase prior to thrombolytic treatment and may predict the risk of hemorrhagic transformation in patients with acute stroke^46^. Several studies have reported that Serum S100B is an early marker of BBB disruption^47^,^48^, but this may have no relevance to neuronal damage^47^.

Similar adverse events including headache, cough, fever. diarrhea, vomiting, constipation, skin puritis, and urinary problems was were observed in the CG and TG in the present study. One patient died because of heart failure, which was unrelated to PG2 administration and involved a history of myocardial infarction. Overall, administration of IV PG2 for two weeks is safe.

The limitations of this study are as follows: (1) a small sample size, (2) a short investigation time of only 3 months after ICH, and (3) a safety evaluation of IV PG2 based on administration only 6 times in 2 weeks. These drawbacks can be overcome by (1) performing a larger study, (2) observing patient follow-up for more than 3 months, and (3) re-evaluating post-PG2 safety more frequently and for a longer duration than in this study.

IV PG2 administration for 2 weeks did not increase the percentage of good GOS and mRS scores, and that also not produced any anti-inflammatory properties. However, these results are preliminary and conducting a more thorough assessment of them is necessary.

## Additional Information

**How to cite this article:** Chen, C.-C. *et al*. PG2 for patients with acute spontaneous intracerebral hemorrhage: a double-blind, randomized placebo-controlled study. *Sci. Rep.* **7**, 45628; doi: 10.1038/srep45628 (2017).

**Publisher's note:** Springer Nature remains neutral with regard to jurisdictional claims in published maps and institutional affiliations.

## Figures and Tables

**Table 1:** Baseline characteristics of demographic data.

Variable	CG (%) (N = 31)	TG (%) (N = 30)	*p*-value
Gender			0.2
Female	11 (35.5)	5 (16.7)	
Male	20 (64.5)	25 (83.3)	
Age (years)	56.4 ± 13.7	53.0 ± 10.1	0.3
Height (cm)	163.7 ± 8.5	165.1 ± 7.5	0.5
Weight (kg)	67.0 ± 20.2	73.4 ± 16.0	0.5
SBP (mmHg)	146.2 ± 22.7	138.5 ± 18.2	0.2
DBP (mmHg)	86.7 ± 20.8	83.7 ± 13.5	0.5
Pulse (times/min)	76.8 ± 12.3	76.8 ± 12.3	1.0
BT (°C)	36.4 ± 0.4	36.6 ± 0.4	0.1
Medical History			
AF			0.5^†^
No	31 (100.0)	29 (96.7)	
Yes	0 (0.0)	1 (3.3)	
IHD			1.0^†^
No	29 (93.5)	29 (96.7)	
Yes	2 (6.5)	1 (3.3)	
HT			0.7^†^
No	4 (12.9)	5 (16.7)	
Yes	27 (87.1)	25 (83.3)	
DM			1.0^†^
No	27 (87.1)	26 (86.7)	
Yes	4 (12.9)	4 (13.3)	
Physical Examination			0.8
No	7 (22.6)	5 (16.7)	
Yes	24 (77.4)	25 (83.3)	
Previous or Ongoing Medications			
Statins			0.5^†^
No	31 (100.0)	29 (96.7)	
Yes	0 (0.0)	1 (3.3)	
Antiplatelets			—
No	31 (100.0)	30 (100.0)	
Yes	0 (0.0)	0 (0.0)	
GCS (score)	15(0)	15(1)	0.18
IVH			
No	31 (100.0)	29 (96.7)	0.52
Yes	0(0.0)	1(3.3)	
Blood Volume (ml^3^)	13.5 ± 8.3	14.2 ± 8.1	0.77
Hemisphere			0.57
Right	17 (55.8)	15 (50.0)	
Left	14 (42.2)	15 (50.0)	

CG: control group; TG: treatment group; SBP: systolic blood pressure; DBP: diastolic blood pressure; BT: body temperature; AF: atrial fibrillation; IHD: ischemic heart disease; HT: hypertension: DM: diabetic mellitus; GCS: Glasgow coma scale presented as median (IQR); IVH: intraventricular hemorrhage; independent test for continuous data; chi-squared test for categorical data; ^†^Fisher’s exact test.

**Table 2:** Effect of PG2 on percentage rate changes between good and bad GOS and mRS in patients with acute spontaneous intracerebral hemorrhage.

Variable (score)	CG (%) (N = 31)	TG (%) (N = 30)	*P*-value
GOS (1–5)			
B			0.7^†^
Bad (1–3)	27 (87.1)	24 (82.8)	
Good (4–5)	4 (12.9)	5 (17.2)	
W1			0.9
Bad (1–3)	22 (71.0)	19 (65.5)	
Good (4–5)	9 (29.0)	10 (34.5)	
W4			0.4
Bad (1–3)	16 (51.6)	11 (37.9)	
Good (4–5)	15 (48.4)	18 (62.1)	
W12			0.2
Bad (1–3)	16 (51.6)	9 (31.0)	
Good (4–5)	15 (48.4)	20 (69.0)	
mRS (0–5)			
B			0.7^†^
Good (0–2)	2 (6.5)	3 (10.3)	
Bad (3–5)	29 (93.5)	26 (89.7)	
W1			1.0
Good (0–2)	6 (19.4)	6 (20.7)	
Bad (3–5)	25 (80.6)	23 (79.3)	
W4			1.0
Good (0–2)	13 (41.9)	11 (37.9)	
Bad (3–5)	18 (58.1)	18 (62.1)	
W12			0.3
Good (0–2)	14 (45.2)	18 (62.1)	
Bad (3–5)	17 (54.8)	11 (37.9)	

GOS: Glasgow outcome scale; mRS: modified Rankin scale; CG: control group; TG: treatment group; Bad: bad outcome, GOS scale score from 1 to 3, mRS scale score from 3 to 5; Good: good outcome, GOS scale score from 4 to 5, mRS scale score from 0 to 2; Differences in categorical variables were tested using the chi-squared test; ^†^Fisher’s exact test.

**Table 3:** Effect of PG2 on CRP, ESR, IL-6, IL-1β, TNF-σ, and S100B in patients with acute spontaneous intracerebral hemorrhage.

Variable	CG (%) (N = 31)	TG (%) (N = 30)	*p*-value^a^
CRP (mg/L)			
B	0.7 ± 0.7	1.2 ± 3.2	0.9
D4	1.8 ± 2.7	2.3 ± 4.2	0.9
D7	1.9 ± 3.3	1.8 ± 2.8	0.9
D14	1.2 ± 3.4	1.2 ± 2.3	0.9
D4-B	1.1 ± 2.6	1.1 ± 3.8	1.0
D7-B	1.2 ± 3.2	0.6 ± 2.6	0.6
D14-B	0.5 ± 3.0	0.1 ± 1.9	0.8
ESR (mm/h)			
B	12.8 ± 9.7	14.9 ± 23.6	1.0
D4	18.0 ± 10.5	19.2 ± 26.1	0.9
D7	20.6 ± 13.1	23.0 ± 27.5	0.8
D14	22.4 ± 18.9	24.3 ± 26.4	0.9
D4-B	5.2 ± 7.6	4.3 ± 8.4	0.8
D7-B	7.8 ± 9.8	8.1 ± 13.1	0.9
D14-B	9.7 ± 13.7	9.3 ± 13.1	0.9
IL-6 (pg/ml)			
B	2.2 ± 3.37	2.2 ± 1.8	1.0
D4	1.9 ± 3.01	3.6 ± 5.9	0.9
D7	7.3 ± 30.2	2.4 ± 2.6	0.8
D14	1.5 ± 2.2	1.9 ± 2.7	0.9
D4-B	−0.3 ± 2.2	1.4 ± 6.2	0.7
D7-B	5.1 ± 30.4	0.2 ± 2.5	0.6
D14-B	−0.7 ± 2.9	−0.3 ± 2.5	0.8
IL-1β (pg/ml)			
B	1.9 ± 1.7	2.7 ± 2.7	0.9
D4	2.0 ± 1.8	2.3 ± 1.9	0.9
D7	2.0 ± 1.7	2.2 ± 1.8	0.8
D14	2.1 ± 2.0	1.9 ± 1.5	0.9
D4-B	0.1 ± 1.4	−0.4 ± 2.7	0.7
D7-B	0.1 ± 1.5	−0.5 ± 2.8	0.6
D14-B	0.2 ± 1.6	−0.9 ± 2.4	0.2
TNF-α (pg/ml)			
B	12.2 ± 29.4	13.3 ± 41.6	1.0
D4	12.8 ± 31.2	11.6 ± 33.2	0.9
D7	24.9 ± 57.8	8.0 ± 20.1	0.5
D14	25.2 ± 53.2	4.9 ± 7.4	0.2
D4-B	0.6 ± 3.2	−1.7 ± 10.1	0.7
D7-B	12.7 ± 49.0	−5.4 ± 22.9	0.4
D14-B	13.1 ± 39.9	−8.5 ± 36.6	0.2
S100B (pg/ml)			
B	79.6 ± 143.0	57.2 ± 62.7	0.9
D4	48.6 ± 55.0	40.4 ± 66.3	0.9
D7	60.1 ± 79.2	35.8 ± 46.7	0.5
D14	43.1 ± 61.0	34.5 ± 51.0	0.9
D4-B	−31.0 ± 137.2	−16.8 ± 87.0	0.8
D7-B	−19.5 ± 112.2	−21.4 ± 80.9	0.9
D14-B	−36.5 ± 146.4	−22.7 ± 83.2	0.8

Data presented as mean ± standard deviations. CG: control group; TG: treatment group; CRP: C-reactive protein; ESR: Erythrocyte sedimentation rate; IL-6: interleukin-6; IL-1β: interleukin-1β; TNF-α: tumor necrosis factor-α; S100B: S100B protein; B: baseline; D4: 4^th^ day after intravenous injection of PG2; D7: 7^th^ day after intravenous injection of PG2; D14: 14^th^ day after intravenous injection of PG2; independent test for continuous data. ^a^corrected p-value for multiple testing.

**Table 4:** The area under the curve (AUC) measures.

Variable	CG (%) (N = 31)	TG (%) (N = 30)	*p*-value
Log CRP			
AUC_1–14_ (day × mg/L)	16.2 ± 8.7	19.5 ± 12.3	0.2
Log ESR			
AUC_1–14_ (day × mm/h)	35.8 ± 7.8	33.6 ± 11.8	0.4
Log IL-6			
AUC_1–14_ (day × pg/ml)	10.2 ± 7.9	13.7 ± 7.1	0.1
Log IL-1*β*			
AUC_1–14_ (day × pg/ml)	11.8 ± 5.6	10.8 ± 5.0	0.5
Log TNF-α			
AUC_1–14_ (day × pg/ml)	24.4 ± 16.8	23.9 ± 15.4	0.9
Log S100B			
AUC_1–14_ (day × pg/ml)	39.4 ± 18.2	40.7 ± 15.8	0.9

CG: control group; TG: treatment group; CRP: C-reactive protein; ESR: Erythrocyte sedimentation rate; IL-6: interleukin-6; IL-1β: interleukin-1β; TNF-α: tumor necrosis factor-α; S100B: S100B protein; AUC 1–14: AUC from baseline to day 14 after PG2 treatment; independent test for continuous data.

**Table 5:** Adverse events following PG2 treatment.

Variable	CG (%) (N = 31)	TG (%) (N = 30)	χ^2^	P value
Adverse event			0.82	0.37
No	14 (45.17)	18 (60.00)		
Yes	17 (54.84)	12 (40.00)		
headache				0.20^†^
No	26 (83.87)	29 (96.67)		
Yes	5 (16.13)	1 (3.33)		
Cough/fever				0.47^†^
No	25 (80.65)	27 (90.00)		
Yes	6 (19.35)	3 (10.00)		
Diarrhea/vomiting				0.11^†^
No	31 (100.00)	27 (90.00)		
Yes	0 (0.00)	3 (10.00)		
Constipation				0.11^†^
No	27 (87.10)	30 (100.00)		
Yes	4 (12.90)	0 (0.00)		
Skin puritis				1.00^†^
No	28 (90.32)	27 (90.00)		
Yes	3 (9.68)	3 (10.00)		
Urinary problem				0.20^†^
No	26 (83.87)	29 (96.67)		
Yes	5 (16.13)	1 (3.33)		

CG: control group; TG: treatment group; independent test for continuous data; chi-squared test for categorical data; ^†^Fisher’s exact test.
